# β-COP Regulates TWIK1/TREK1 Heterodimeric Channel-Mediated Passive Conductance in Astrocytes

**DOI:** 10.3390/cells11203322

**Published:** 2022-10-21

**Authors:** Seong-Seop Kim, Yeonju Bae, Osung Kwon, Seung-Hae Kwon, Jong Bok Seo, Eun Mi Hwang, Jae-Yong Park

**Affiliations:** 1 School of Biosystems and Biomedical Sciences, College of Health Sciences, Korea University, Seoul 02841, Korea; 2 Center for Functional Connectomics, Korea Institute of Science and Technology (KIST), Seoul 02792, Korea; 3 Seoul Center, Korea Basic Science Institute (KBSI), Seoul 02841, Korea

**Keywords:** β-COP, protein–protein interaction, TREK1, TWIK1, astrocytes, passive conductance

## Abstract

Mature astrocytes are characterized by a K^+^ conductance (passive conductance) that changes with a constant slope with voltage, which is involved in K^+^ homeostasis in the brain. Recently, we reported that the tandem of pore domains in a weak inward rectifying K^+^ channel (TWIK1 or KCNK1) and TWIK-related K^+^ channel 1 (TREK1 or KCNK2) form heterodimeric channels that mediate passive conductance in astrocytes. However, little is known about the binding proteins that regulate the function of the TWIK1/TREK1 heterodimeric channels. Here, we found that β-coat protein (COP) regulated the surface expression and activity of the TWIK1/TREK1 heterodimeric channels in astrocytes. β-COP binds directly to TREK1 but not TWIK1 in a heterologous expression system. However, β-COP also interacts with the TWIK1/TREK1 heterodimeric channel in a TREK1 dependent manner and enhances the surface expression of the heterodimeric channel in astrocytes. Consequently, it regulates TWIK1/TREK1 heterodimeric channel-mediated passive conductance in astrocytes in the mouse brain. Taken together, these results suggest that β-COP is a potential regulator of astrocytic passive conductance in the brain.

## 1. Introduction

Astrocytes constitute the largest portion of cells and are extremely dense in the brain [1]. Unlike neurons, which are electrically activated and receive signals through synapses, astrocytes extend processes between the synaptic gaps of neurons and directly interact with pre- and postsynaptic neurons, thereby regulating the formation, function, and elimination of synapses [2]. In particular, astrocytes form a gap junction that is densely and closely connected between cells. This enables the movement of ions, water, glutamate, and gamma-aminobutyric acid (GABA) between cells, and plays an important role in regulating physiological homeostasis in the brain [3,4,5].

All cells have a constant membrane potential maintained by various ions in the cell. This membrane potential becomes a source of energy to operate the channel, especially in excitatory neurons or muscle cells, enabling signal transmission between cells. The membrane potential is similar to the equilibrium potential of potassium and changes sensitively in response to the potassium concentration outside the cell [6]. This occurs because the cell membrane has very high potassium permeability due to the high density of potassium channels such as Kir4.1 and leak K^+^ channels [7]. Potassium above an appropriate level in the brain causes depolarization of the resting neuronal membrane potential and affects a variety of neuronal activity [4]. Perturbations in potassium homeostasis can induce neurological diseases such as seizures and ataxia [4,6]. Therefore, maintaining potassium homeostasis is essential for the central nervous system. Although the exact mechanism of potassium homeostasis in the brain remains ambiguous, many studies have shown that astrocytes may play an important role in homeostasis maintenance.

The excess potassium accumulated outside nerve cells is taken up by the channels in astrocyte membranes, transferred to astrocytes with a low concentration of extracellular potassium through the gap junction and released [4,8,9]. This process is called potassium clearance, and contributes to the maintenance of potassium homeostasis [4]. Disruption of this process in astrocytes causes neurological diseases including epilepsy. Potassium clearance by astrocytes allows for a linear current–voltage (*I*–*V*) relationship that differentiates them from other cells, which is called passive conductance [10]. The physiological regulatory mechanism of passive conductance remains unclear but is considered an important characteristic resulting from intrinsic astrocyte functions.

The tandem of pore domains in a weak inward rectifying K^+^ channel (TWIK1, K2P 1.1, or KCNK1) and TWIK-related K^+^ channel 1 (TREK1, K2P 2.1, or KCNK2) are members of the two pore domain potassium (K2P) channel family, consisting of 15 channels that regulate the stabilization of resting membrane potential and cellular excitability by wielding background K^+^ leakage currents [11]. Although TWIK1 is considered a non-functional channel, recent studies showed that TWIK1 can act as a heterodimeric channel [12]. The TWIK1/TASK3 heterodimeric channel regulates the intrinsic excitability of dentate granule neurons [13]. Recently, we reported that TWIK1 and TREK1 form TWIK1/TREK1 heterodimeric channels in a disulfide bond-dependent manner. Additionally, these channels are the major molecular identities that constitute the passive conductivity of hippocampal astrocytes and regulate glutamate release [14]. However, the functional regulatory mechanisms of these heterodimeric channels remain poorly understood.

Among the components of TWIK1/TREK1 heterodimeric channels, the regulatory mechanisms of TREK1 have been well studied. TREK1 is a polymodal activated channel regulated by a wide variety of chemical and physiological factors [15]. The activity is regulated by intracellular acidosis, a stretch of mechanical stimulation, polyunsaturated fatty acids (PUFA) such as omega3, general anesthesia [16], protein kinase A (PKA), and protein kinase C (PKC). Furthermore, its expression is disturbed in pathological conditions such as depression or ischemia [17,18,19,20]. TREK1 contains various binding proteins such as microtubule-associated protein 2 (Mtap2), A-kinase anchoring protein 150 (AKAP150) [21], and Spadin [22]. β-coat protein (COP) is a subunit of the COP1 complex involved in protein transport between the Golgi apparatus and endoplasmic reticulum (ER) [23]. We reported that β-COP regulates membrane trafficking by directly binding to TREK1 [24]. Compared to TREK1, regulation mechanisms and binding proteins of TWIK1 remain poorly understood. Functional properties of K2P channels are regulated by a variety of intracellular proteins for single conductivity, open probability, and membrane transport [25,26]. Therefore, TWIK1 or TREK1 binding proteins may also regulate the functions of TWIK1/TREK1 heterodimeric channels, although intracellular TWIK1 binding proteins have not yet been reported.

To test this hypothesis, we examined the role of β-COP on the function of TWIK1/TREK1 heterodimeric channels. In this study, we found that β-COP interacts with the TWIK1/TREK1 heterodimeric channel in a TREK1-dependent manner and enhances passive conductance in astrocytes. We also identified the exact binding motifs of TREK1 that are recognized by β-COP. These results clearly demonstrate that β-COP is a pivotal binding protein of TWIK1/TREK1 heterodimeric channels, which regulates astrocytic passive conductance in the brain.

## 2. Materials and Methods

### 2.1. Chemical

Spadin was obtained from Peptron (Peptide Custom Service, Daejeon, Korea). Stock solutions of all materials were stored at −20 °C and diluted in water to the required concentration immediately before the experiment.

### 2.2. Genetic Material and Vectors Used in Experiments

cDNAs encoding the full-length mouse TREK1 isotype 2 (GenBank accession no. NM_010607), TREK1 isotype 1 (NM_001159850), mouse TWIK1 (NM_008430), and mouse β-COP (NM_033370) cDNAs were generated by reverse transcription polymerase chain reaction (RT-PCR). Several mutants of TREK1 including TREK1ΔN1, TREK1ΔN1,2, TREK1ΔN1,3, TREK1ΔN1,4, and TREK1ΔN4 were generated using mouse TREK1 isotype 2 cDNA as a template using an EZchangeTM site-directed mutagenesis kit (Enzynomics). All expression vectors used in the experiment were cloned into various destination vectors, including pDEST-FLAG-C, pDEST-EGFP-N, and pDEST-IRES2-GFP using the gateway cloning method. TWIK1 shRNA and TREK1 shRNA were constructed for gene silencing in cultured astrocytes and have been previously validated for their effectiveness. The target regions of shRNA were as follows: mouse TWIK1: 5’-GCATCATCTACTCTGTCATCG-3’; mouse TREK1: 5’-GCGTGGAGATCTACGACAAGT-3’; mouse β-COP: 5’-GCAGTTAGCACTGGATCTTGT-3’; mouse α-COP: 5’- atagttaccctaatcgcaatt-3’; mouse γ-COP: 5’- gctcgggtctttaacgaaact-3’; and mouse ADP-ribosylation factor 1 (Arf1): 5’- gatgctgttctcttggtgttt-3’. pSicoR β-COP, α-COP, γ-COP, and Arf1 shRNAs were constructed using the EZchangeTM site-directed mutagenesis kit (Enzynomics) and confirmed by fluorescence imaging and immunoblotting. Co-transfection with the two types of shRNAs was accomplished using different pSicoR vectors expressing mCherry and GFP fluorescent proteins.

### 2.3. Primary Astrocyte Culture and Electroporation

Primary cortical astrocytes were obtained from 1-day-old male and female f C57BL/6 mouse pups. The isolated cerebral cortex was dissociated into a single cell suspension by gentle pipetting after the meninges were removed. The resulting single cell suspension was plated on glass coverslips coated with 0.1 mg/mL poly D-lysine (PDL) and used four days later. Cells were grown in Dulbecco’s modified Eagle’s medium (DMEM, Invitrogen) supplemented with 10% heat-inactivated fetal bovine serum, 10% heat-inactivated horse serum, and 100 units per mL of penicillin-streptomycin. Cell plates were maintained at 37 °C in a humidified 5% CO_2_ incubator. On day four of incubation, the cells were gently washed to remove other suspended cell types and debris. Occasionally, the purity of cultured astrocytes was monitored by immunocytochemical staining with a specific antibody against glial fibrillary acidic protein (GFAP). Above 95% of primary cultured cells normally showed GFAP-positive signals. On the following day (day five of incubation), cells were placed on glass coverslips coated with PDL, and an optimized voltage protocol was used (Thermo Fisher, Waltham, MA, USA) using a Neon Electroporation instrument (Thermo Fisher, Waltham, MA, USA). A total of 1200 V, 20 ms pulse width, and 2 pulse counts were used to transfect cultured astrocytes with various shRNAs.

### 2.4. Cell Lines and Transfection

Human embryonic kidney (HEK) 293T and COS7 cells were purchased from the Korea Cell Line Bank (Seoul National University, Seoul, Korea). Cells were grown at 37 °C in Dulbecco’s modified Eagle’s medium (DMEM, Invitrogen) added with 10% fetal bovine serum (Invitrogen) and 100 units per mL of penicillin-streptomycin. The humidified atmosphere consisted of 95% air and 5% CO2. Cells were transfected with polyetherimide (PEI; Sigma-Aldrich).

### 2.5. Immunocytochemistry (ICC)

Cultured astrocytes on coverslips were fixed in 4% paraformaldehyde (PFA) at room temperature for 20 min and permeabilized with phosphate buffered saline (PBS) containing 0.5% NP-40 for 10 min. Non-specific binding caused by the antibody was prevented by incubation for 2 h in 3% donkey serum (GeneTex). Thereafter, cells were incubated at 4 °C overnight in donkey serum-containing anti-TREK1 (Alomone Labs, # APC-047, 1: 200) and anti-β-COP (Santa Cruz Biotechnology; sc-393615, 1: 200). After washing the next day, Dye Light 488, 549 conjugated secondary antibodies (Jackson Labs, 1:500) were treated and incubated at room temperature for 1 h. After that, the cells were mounted, and observed under a Nikon A1 confocal microscope to determine the surface expression of TREK1 according to the decrease in β-COP expression. Anti-TREK1 was used in cultured astrocytes transfected with β-COP shRNA in the same manner as above. Before permeation, plasma membrane staining was performed with WGA-647 conjugate (1: 200; W32466, Thermo) through additional culture at 4 °C for 15 min. 

### 2.6. Duolink Proximity Ligation Analysis

A Duolink Proximity ligation assay (PLA) was performed using anti-TREK1 (Alomone Labs, # APC-047, 1:100) or anti-TWIK1 antibody (Santa Cruz Biotechnology; sc-11483, 1:100) with β-COP (1:200; D- 10, Santa Cruz Biotechnology) using Bioscience’s in situ PLA kit according to the manufacturer’s instructions. The PLA probe anti-rabbit minus bound to anti-TREK1 and TWIK1 antibodies, and the PLA probe anti-mouse plus bound to anti-β-COP antibodies. Cultured astrocytes were transfected with scrambled (Sc) or β-COP shRNA and incubated for 24 h before the experiments. The cells were observed under a Nikon A1 confocal microscope.

### 2.7. Co-Immunoprecipitation (Co-IP) and Immunoblotting

For Co-IP, cultured astrocytes and HEK293T cells were washed with PBS and then lysed with RIPA buffer composed of 50 mM Tris-Cl, 150 mM NaCl, 1% NP-40, 0.5% sodium deoxycholate, 0.1% sodium dodecyl sulfate (SDS), and protease inhibitor cocktail (Tech & Innovation, Kangwon, Korea), and incubated for 1 h at 4 °C. Then, the lysate was centrifuged at 13,000× *g* for 20 min at 4 °C, and only the supernatant was transferred to a new tube. For co-IP, the reaction was carried out overnight at 4 °C on a rocking mixer with the corresponding antibody (anti-TREK1, Alomone Labs; anti-β-COP, Santa Cruz Biotechnology; anti-Flag, Sigma-Aldrich). Subsequently, the reaction was mixed with Protein G Agarose (Santa Cruz Biotechnology) for 1 h and gently washed three times with LIPA buffer. For immunoblotting, protein samples were separated by SDS–polyacrylamide gel electrophoresis (PAGE) using 10% gels. The separated proteins on the gel were transferred onto polyvinylidene fluoride membranes. The blots were incubated overnight at 4 °C with an anti-TREK1 antibody (Alomone Labs; 1:1000), anti-FLAG antibody (Sigma-Aldrich, 1:1000), or anti-β-COP antibody (Santa Cruz Biotechnology, 1:500) anti-GFP antibody (Santa Cruz Biotechnology, 1:1000). Blots were then washed and incubated with horseradish peroxidase-conjugated goat anti-mouse, goat anti-rabbit, or anti-rabbit IgG, followed by washing and detection of immunoreactivity using enhanced chemiluminescence (Amersham Biosciences).

### 2.8. Real-Time Quantitative Reverse Transcription-Polymerase Chain Reaction (qRT-PCR)

RNA was isolated from cultured mouse astrocytes according to the manufacturer’s instructions using an RNA purification kit (Geneol, Korea). As soon as RNA was extracted, cDNA was synthesized using the SuperScript VILO cDNA Synthesis Kit (Invitrogen) according to the manufacturer’s instructions. The SYBR^®^ Select Master Mix (Invitrogen, New York) was used, and qRT-PCR was performed using a Step One Plus instrument (Life Technologies, New York). Each assay was performed on astrocytes cultured from different mice. Glyceraldehyde 3-phosphate dehydrogenase (GAPDH) was used as an internal reference and run in parallel with the target gene. Data were obtained as cycle threshold (Ct) values (strike cycles). The expression level of the target gene was expressed as 2-ΔCT, where ΔCT represents the difference in Ct between the gene of interest and GAPDH.

### 2.9. Bimolecular Fluorescence Complementation (BiFC) Experiment

For BiFC analysis, TREK1 isotype 1, TREK1 isotype 2, and all deficient mutants of TREK1 were cloned into all possible combinations of bimolecular fluorescence complement (pBiFC)- VN173 and pBiFC-VC155 vectors. Vectors containing each of the two genes for the binding test were transfected into cells and fixed with 4% paraformaldehyde for 20 min at room temperature, 24 h after transfection. The nuclei were then stained with DAPI for 3 min and mounted with Dako fluorescence mounting medium. Fluorescence was observed using a confocal microscope.

### 2.10. Electrophysiological Recording

For electrophysiological data, cultured astrocytes were plated on coverslips 24 h after transfection with the target gene, and COS7 cells were plated on coverslips 12 h after transfection. To measure currents, cells were immersed in a standard bath solution containing: 150 mM NaCl, 3 mM KCl, 2 mM CaCl_2_, 1 mM MgCl_2_, 10 mM N-2-Hydroxyethylpiperazine-N’-2-Ethanesulfonic Acid (HEPES), 5.5 mM D-glucose, and 20 mM sucrose (pH 7.4, adjusted with NaOH). A patch pipette was made using a borosilicate glass capillary (Warner Instruments, Washington, DC, USA), and filled with a standard solution containing:150 mM KCl, 1 mM CaCl_2_, 1 mM MgCl_2_, 5 mM EGTA, and 10 mM HEPES (pH 7.2, adjusted with KOH). A Digidata 1550 A interface (Axon Instruments, Union City, CA, USA) was used to convert the digital-to-analog signals between the computer and amplifier, and Clampfit software (Axon Instruments, Union City, CA, USA) was used to analyze the currents. The current−voltage (*I*−*V*) curves were measured by applying 1-s ramp pulse (from −150 mV to +50 mV) from a holding potential of −60 mV while continuously perfusing the bathing solution into the chamber (RC-25 chamber, Warner Instruments, Washington, DC, USA) at a rate of 1 mL/min. Data was sampled at 5 kHz and filtered at 1 kHz. All experiments were performed at room temperature of 20−22 °C.

### 2.11. Immunohistochemistry (IHC)

Brain slices were washed with PBS at room temperature for 20 min, followed by antigen retrieval at 85 °C for 30 min using 10 mM sodium citrate buffer. Slices were permeabilized with 0.4% Triton X-100 in PBS at RT for 20 min. The slices were then blocked with 10% donkey serum and 0.1% Triton X-100 in PBS at RT for 3 h, followed by incubation with primary antibodies, 5% donkey serum, and 0.1% Triton X-100 in PBS at 4 ◦C overnight. After washing three times with 0.1% Triton X-100 in PBS at RT for 15 min, secondary antibodies, 5% donkey serum, and 0.1% Triton X-100 in PBS were added for 2 h at 4 °C. The slices were counterstained with DAPI and mounted with a mounting medium (Vectashield, Vector Laboratories Inc., Burlingame, CA, USA). The following antibodies were used: rabbit anti-TREK1 (Alomone Labs, Jerusalem, Israel, 1:200; RRID: APC-047, 1:200), mouse anti-β-COP (Santa Cruz Biotechnology, D-10, 1:200), rat anti-GFAP (Thermo Fisher, Waltham, MA, USA; 1:500, RRID:13-0300, 1:500), and Alexa Fluor 488-, 594-, and 647-conjugated secondary antibodies (Jackson ImmunoResearch, West Grove, PA, USA; 1:300). All images were acquired using a Nikon Ti2 confocal microscope (Nikon Instruments, Inc., Melville, NY, USA).

### 2.12. Electrophysiological Recording in Hippocampal Slices

The CA1 region of the hippocampus was sliced (350 mm) using a vibrating blade microtome (Leica VT1000 S) 55 into the brains of virus-infected mice. One prepared slice was recovered in oxygenated (95% O_2_ and 5% CO_2_) artificial cerebrospinal fluid (130 mM NaCl, 24 mM NaHCO_3_, 3.5 mM KCl, 1.25 mM NaH_2_PO_4_, 1.5 mM CaCl_2_, 1.5 mM MgCl_2_, and 10 mM glucose saturated with 95% O_2_–5% CO_2_ at pH 7.4) for 1 h. Hippocampal slices were incubated in a chamber containing artificial cerebrospinal fluid (ACSF) consisting of 130 mM NaCl, 24 mM NaHCO_3_, 3.5 mM KCl, 1.25 mM NaH_2_PO_4_, 1.5 mM CaCl_2_, 1.5 mM MgCl_2_, and 10 mM glucose 95% O_2_–5% CO_2_ was continuously supplied during the experiment. Additionally, to determine the localization of astrocytes in the hippocampus, brain slices were treated with SR-101 (Sigma; 1 μM) dye for 30 min prior to current measurement. The patch pipette was filled with a solution containing 140 mM KCl, 10 mM HEPES, 5 mM EGTA, 2 mM Mg-ATP and 0.2 mM Na-GTP adjusted to pH 7.4 with KOH. Axopatch 200 A (Axon Instruments, Union City, CA, USA) was used to perform whole-cell patch recordings of astrocytes in the hippocampus. Whole-cell currents were recorded after applying 1-s voltage steps from −160 mV to +40 mV in 10 mV increments from a holing potential of −60 mV.

### 2.13. Statistics

Analysis of the experiment was performed using the Clampfit and SigmaPlot software. Images analyzed by confocal microscopy were analyzed in Image J, and quantification of all data was expressed as mean ± standard error of the mean (SEM). Significance was evaluated using Student’s *t*-test (paired t-test) or one-way ANOVA followed by Tukey’s post hoc test, and the significance levels were specified as follows. N.S: not significant, * *p* < 0.05, ** *p* < 0.01, and *** *p* < 0.001. Prism9.0 software (GraphPad Software, San Diego, CA, USA) was used for carrying out the statistical analysis.

## 3. Results

### 3.1. β-COP Is Associated with Endogenous TREK1 in Cultured Astrocytes

**Figure 1:**
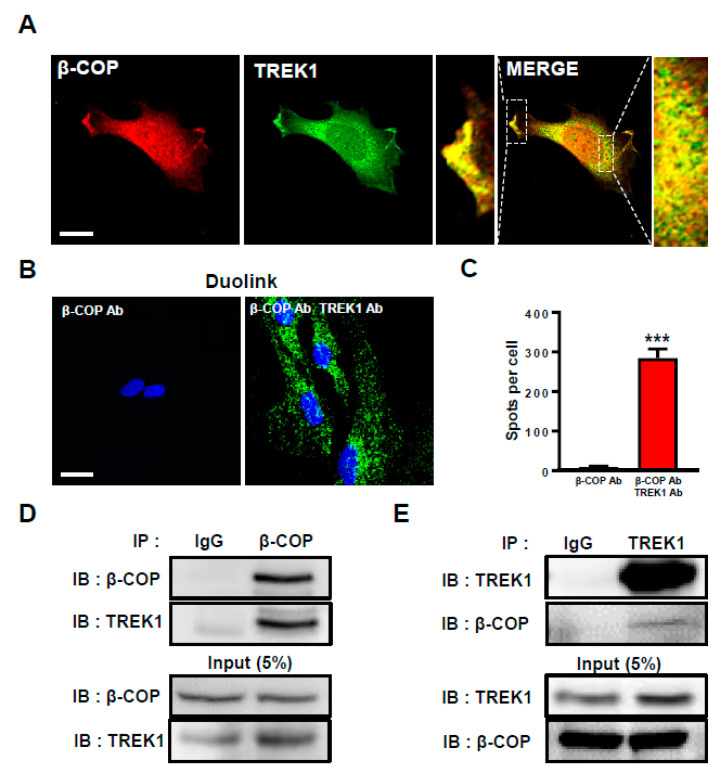
β-COP associates with TREK1 in astrocytes. (**A**) Representative immunocytochemical images of astrocytes stained with anti-β-COP and anti-TREK1 antibodies. Enlarged images on either side of the merged image indicate co-localization of β-COP and TREK1 in the plasma membrane and inside of astrocytes. Scale bar, 20 µm. (**B**) Duolink PLA assay. Intense PLA signals were detected in anti-β-COP antibody and anti-TREK1 antibody exposed astrocytes, but not in anti-β-COP only exposed astrocytes. Scale bar, 20 µm. (**C**) The PLA signals were counted with Image J software and the average number of spots per cell is presented in the graph. The number at the bottom of each bar indicates n. (**D**) Co-immunoprecipitation (Co-IP) assay in astrocytes. Whole-cell lysates were immunoprecipitated using anti-TREK1 and then blotted using anti-β-COP. (**E**) The reverse is immunoprecipitated using anti-β-COP and then blotted using anti-TREK1. All values are mean ± SEM (*** *p* < 0.001). SEM, standard error of the mean.

Previously, we reported that β-COP binds directly to TREK1 in a heterologous expression system [24]. To investigate the protein–protein binding between β-COP and TREK1 in astrocytes, we first examined the subcellular localization of both proteins in astrocytes. Anti-TREK1 and anti-β-COP were used to perform double staining ICC of cultured astrocytes. Strong overlapping signals were observed (Figure 1A). TREK1 and β-COP colocalized at the membrane and subcellular regions in the enlarged merged image. To determine whether TREK1 and β-COP had an endogenous protein–protein interaction, we performed a Duolink proximity ligation assay (PLA). When only anti-β-COP was used, the signal was not observed; however, when anti-TREK1 and anti-β-COP were combined, strong Duolink PLA signals were observed (Figure 1B). The bar graph shows clear differences in PLA signals (Figure 1C). To supplement these results, we performed co-IP. In the sample immunoprecipitated with β-COP, we observed a strong signal (Figure 1D). Similarly, we detected a clear signal in reverse co-IP (Figure 1E). Taken together, these data suggest that β-COP and TREK-1 were endogenous and strongly associated in cultured astrocytes.

### 3.2. β-COP Interacts with TREK1 Isoforms

**Figure 2:**
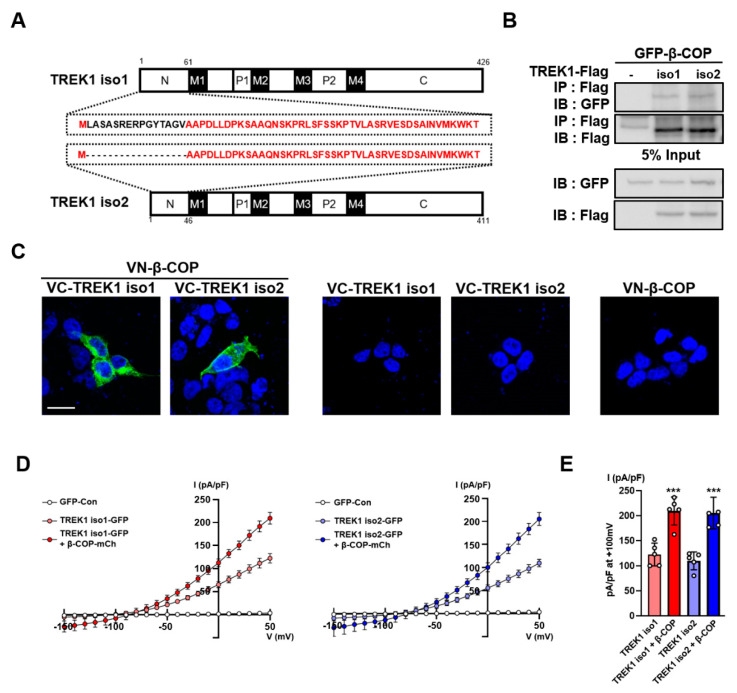
β-COP interacts with TREK1 isoforms. (**A**) Schematic of full-length TREK1 isoforms, TREK1 isotype 1 (up), TREK1 isotype 2 (down), and identical amino acids are marked in red. (**B**) Co-IP in HET293T cell. After transfection of Flag-tagged isoforms of TREK1 and GFP-β-COP, immunoprecipitation with Flag was performed and blotted with GFP. (**C**) The bimolecular fluorescence complementation (BiFC) assay in HET293T cells. Venus fluorescence was observed when VN-β-COP and VC-TREK1 isotype 1 or VC-TREK1 isotype 2 were co-transfected and was not when transfected alone. Scale bar, 20 µm. (**D**) Averaged current-voltage (*I*-*V*) relationship of GFP-Con transfected(white), TREK1 isotype 1 IRES2 GFP transfected (light red), TREK1 isotype 1 IRES2 GFP and β-COP IRES2 mCherry co-transfected (red) astrocytes (left), GFP-Con transfected (white), TREK1 isotype 2 IRES2 GFP transfected (light blue), TREK1 isotype 2 IRES2 GFP, and β-COP IRES2 mCherry co-transfected (blue) astrocytes (right). (**E**) Bar graph indicated TREK1-mediated currents averaged from results in (**D**) at +50 mV. All values are expressed as the mean ± SEM. SEM, standard error of the mean. *p*-values were obtained with Student’s *t*-test. *** *p* < 0.001.

TREK1 has two isoforms that are identical but differ only in the exon1 sequence. They are identical for only a fraction of EXON1, which differs in the N-terminal amino acid sequences, resulting in amino acid chains of different lengths. The 426 amino acids of TREK1 are TREK1 isotype 1 (NCBI accession number NM_001159850), and the 411 amino acids of TREK1 are TREK1 isotype 2 (NCBI accession number NM_010607) (Figure 2A). To confirm which isoforms are more highly expressed in astrocytes, we performed qPCR analysis using primer sets created using different exon1 sequences of TREK1 (Figure S1A). The expression level of TREK1 isotype 2 was significantly higher than that of TREK1 isotype 1 (Figure S1B). To determine the difference in the interactions between the two isoforms of TREK1 and β-COP in mammalian systems, we constructed expression vectors for green fluorescent protein (GFP)-tagged β-COP and Flag-tagged TREK1 isoforms. They were transfected into HET293T cells, followed by co-IP with anti-Flag and blotting with anti-GFP. From these data, we found that β-COP binds to the two isoforms of TREK1 and is more strongly associated with TREK1 isotype 2 than with TREK1 isotype 1 (Figure 2B). We also performed bimolecular fluorescence complementation (BiFC) to determine the interactions between TREK1 isoforms and β-COP. In this experiment, we fused the N-terminal- and C-terminal portions of the Venus fluorescence protein into two proteins. If the two proteins interact, the N-terminal- and C-terminal parts fused to the proteins are bound, and complete Venus fluorescence can be expected. We fused the N-terminal portion of Venus to β-COP (VN-β-COP) and the C-terminal portion of Venus to the TREK1 isoforms (VC-TREK1 isotype 1 and VC-TREK1 isotype 2). Strong Venus fluorescence was observed in co-transfected cells (VN-β-COP + VC-TREK1 isoforms) (Figure 2C). In the negative control experiment, the divided Venus-tagged β-COP and TREK1 isoforms were transfected into cells alone, and no fluorescence was observed. TREK1 exhibits a large outward rectifying current when transfected into cells [24]. Next, we measured the contribution of β-COP to the channel activity of TREK1 isoforms using a whole-cell patch-clamp test. We constructed a GFP-tagged TREK1 isoform expression vector and an mCherry-tagged β-COP expression vector and transfected them into Cos7 cells. We observed large K^+^ currents in cells transfected with each TREK1 isoform compared to the control, and K^+^ currents were almost doubled by co-transfection with β-COP. (Figure 2D,E).

### 3.3. Identification of the β-COP Binding Region within the TREK1 Channel

**Figure 3:**
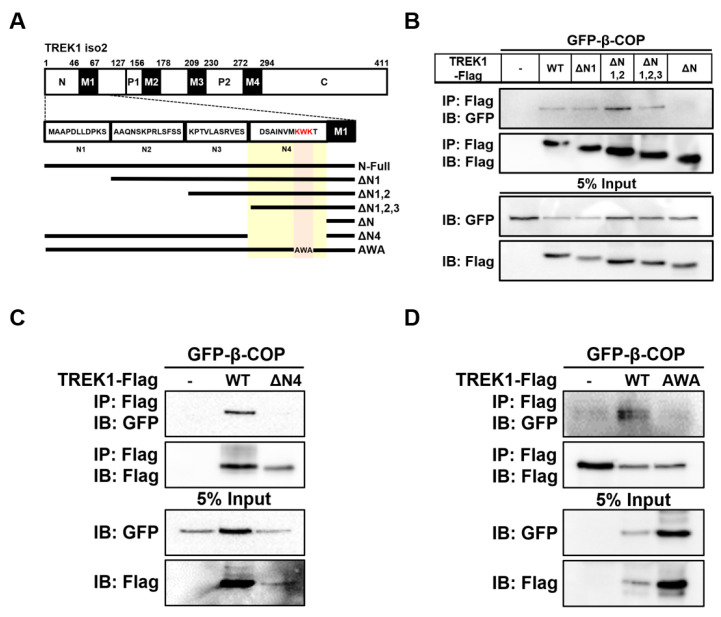
Identification of TREK1 binding site with β-COP. (**A**) Schematic of full-length TREK1 and its N-terminus deletion mutants. (**B**) Co-IP assay in HET293T cell. After transfection of the Flag-tagged TREK1Δ1, TREK1Δ1-2, TREK1Δ1-3, TREK1ΔN4, and GFP-β-COP, immunoprecipitation with the Flag was performed, and blotted with GFP. (**C**) Co-IP assay in HET293T cell. After transfection of the Flag-tagged TREK1WT, TREK1ΔN4, and GFP-β-COP, immunoprecipitation with the Flag was performed and blotted with GFP. Co-IP assay shows β-COP does not interact with only TREK1ΔN4 deletion mutation. (**D**) After transfection of the Flag-tagged TREK1 or TREK1 AWA and GFP-β-COP, immunoprecipitation with the Flag was performed and blotted with GFP.

In our previous study, we reported that β-COP binds directly to the N-terminal region of TREK1 [24]. To determine the binding site of β-COP within TREK1 in more detail, we divided the N-terminal part of TREK1 into four sections and constructed TREK1 N-terminal deletion forms (ΔN1; ΔN1,2; ΔN1,2,3; ΔN) (Figure 3A). Based on the co-IP results showing an association between GFP-β-COP and Flag-TREK1 (Figure 2B), we constructed each expression vector tagged with a Flag in all TREK1 N-terminal deletion forms. The interaction between the TREK1 N-terminus deletion forms and β-COP was investigated using co-IP (Figure 3B). Strong signals were detected for TREK1ΔN1, TREK1ΔN1,2, and TREK1ΔN1,2,3 with β-COP but not for TREK1ΔN with β-COP. Therefore, we predicted that β-COP interacts with the N4 region of TREK1, and thus produced a vector in which only the N4 region was deleted from the N-terminus of TREK1 (TREK1NΔ4) (Figure 3A). As predicted, TREK1NΔ4 showed no signal in the co-IP assay (Figure 3C). Previous studies have shown that β-COP recognizes and binds through conserved motifs such as KXKXX and KKXX [27,28]. We found a KXKXX motif in the N4 region of TREK1 (Figure 3A). To test whether β-COP binds to TREK1 by recognizing the KXKXX sequence at the end of the N-terminal region of TREK1, we constructed an AWA mutant of TREK1 by substituting AXA for KXK in TREK1 using mutagenesis cloning. When substituting KXK for AXA in TREK1, no signal of the interaction of the two proteins was detected (Figure 3D). Taken together, these data suggest that β-COP directly binds to the KWK sequence, the di-lysine motif, in the N-terminal region of TREK1.

### 3.4. β-COP Silencing Decreases TREK1 Surface Expression in Astrocytes

**Figure 4:**
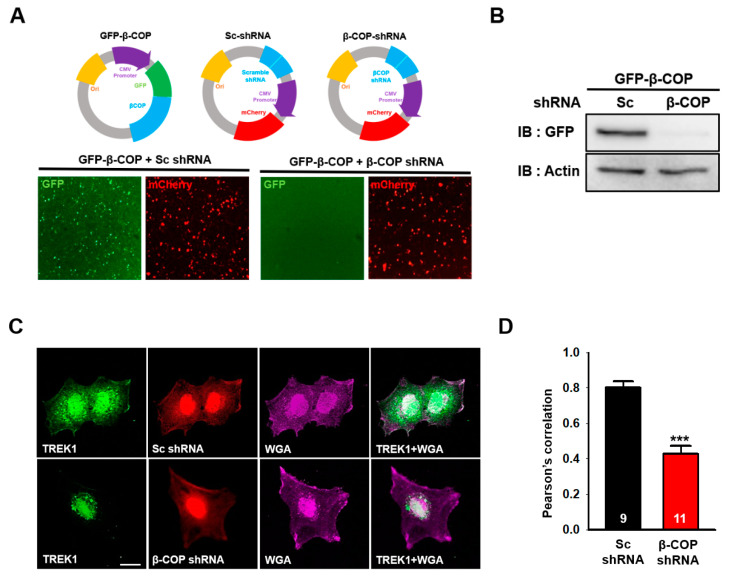

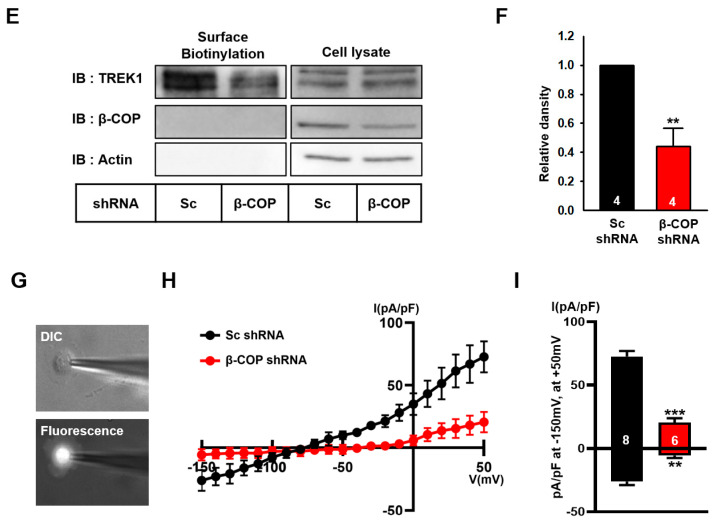
Silencing of β-COP decreases TREK1 trafficking in astrocytes. (**A**) Schematic diagram of the GFP-βCOP, Sc-shRNA, and β-COP shRNA construct. The efficiency of β-COP shRNA was assessed by (down) fluorescent imaging. The shRNA constructs also express an mCherry signal. Scale bar, 100 µm. (**B**) Efficiency of gene silencing by the β-COP shRNA construct was monitored by Western blot. (**C**) Representative immunocytochemistry images stained with the anti-TREK1 antibody in cultured astrocytes transfected with Sc-shRNA or β-COP shRNA. Wheat germ agglutinin (WGA) was used to stain the plasma membrane of astrocytes. Scale bar, 20 µm. (**D**) Pearson’s correlation coefficient for TREK-1 and WGA. (**E**) Cell surface biotinylation assay in Sc shRNA and β-COP shRNA transfected astrocytes. Surface expression of TREK1 was slightly decreased by β-COP shRNA expression. (**F**) Relative density of the cell surface expression. (**G**) DIC(differential interference contrast) (upper) and fluorescence (lower) image of cultured astrocyte transfected with β-COP shRNA. The β-COP shRNA construct also expresses an mCherry signal. (**H**) Average *I*-*V* relationship of Sc shRNA (black) and β-COP shRNA (red) transfected astrocytes. The voltage ramp-induced whole-cell current traces were recorded from each cell. (**I**) Summary bar graph plotted from the results in (H) at +50 mV. All values in (**D**), (**F**), and (**I**) are expressed as the mean ± SEM. SEM, standard error of the mean. *p*-values were obtained with Student’s *t*-test. ** *p* < 0.01, *** *p* < 0.001.

In astrocytes, TREK1 is usually present on the cell membrane and functions as a channel protein [6]. Next, we determined the effect of β-COP on endogenous TREK1 in astrocytes. A short hairpin RNA(shRNA) capable of selectively knocking down the corresponding target mRNA was used for gene silencing [29]. We constructed a specific shRNA complementary to the β-COP mRNA sequence to reduce β-COP expression in astrocytes. The prepared shRNA vector exhibited red fluorescence for recognition and was co-transfected with GFP-β-COP expressing green fluorescence (Figure 4A, upper panel). In the image of the cells co-transfected with GFP-β-COP and β-COP shRNA, the cells expressing green fluorescence almost disappeared (Figure 4A, bottom). In addition, Western blot results showed that β-COP shRNA efficiently knocked down the β-COP protein (Figure 4B). To test the changes in the intracellular localization of TREK-1 under β-COP deficiency in astrocytes, we performed ICC analysis. Cultured astrocytes transfected with Sc shRNA or β-COP shRNA were treated with anti-TREK1 and wheat germ agglutinin (WGA), a plasma membrane marker. In the control, Sc shRNA-transfected astrocytes and endogenous TREK1 proteins were expressed throughout the cell, especially on the membrane surface. However, when β-COP was knocked down with β-COP shRNA, TREK1 proteins were mainly localized in the intracellular regions, and colocalization with WGA was significantly reduced (Figure 4C). It was quantitatively confirmed by Pearson’s correlation coefficient that the expression of TREK1 was significantly reduced on the membrane surface with reduced β-COP expression (Figure 4D). Subsequently, we performed a surface biotinylation assay to quantitatively determine changes in the surface expression level of TREK1 under β-COP deficiency in cultured astrocytes. The total expression level of TREK1 did not change, but the surface expression level of TREK1 was effectively reduced in β-COP shRNA-transfected astrocytes (Figure 4E,F). Since TREK1 is the major channel responsible for the background K^+^ current in astrocytes [14], we measured the astrocytic K^+^ current when the surface expression of TREK1 was decreased by β-COP deficiency. The fluorescence (Figure 4G) confirmed that shRNA functioned adequately in astrocytes. Compared to control Sc shRNA, β-COP shRNA significantly reduced outward and inward currents in astrocytes (Figure 4H,I). In addition, β-COP shRNA treatment showed a positive shift from −77.1 ± 4.0 mV to −61.6 ± 5.6 mV in reversal membrane potential (RMP) of cultured astrocytes, which suggested that the membrane potential of astrocytes is depolarized by the deficiency of β-COP. Overall, β-COP depletion reduced the surface expression of TREK1 and the linear current-voltage (*I*–*V*) relationship in astrocytes.

### 3.5. Other Subunits of the COP1 Complex Do Not Affect the K^+^ Current in Astrocytes

β-COP is a coatomer of the COP1 complex [30]. COP1 is responsible for packaging and transporting various proteins between the endoplasmic reticulum and Golgi apparatus [31]. COP1 is composed of seven different coatomers divided into two groups (coat complex; α β’ε, coat adapter complex; β γ δ ζ) and their assembly is accomplished by the small GTPase Arf1 [23,28,32]. Since β-COP single knockdown was sufficient to regulate the expression of TREK1, we wondered whether β-COP worked alone or acted as a coatomer with other subunits in astrocytes. To test the current-regulating function of astrocytes for different subunits of COP1, we selected α-COP, γ-COP, and Arf1. α- and γ-COP belong to two subgroups, COP1 and Arf1, which are essential components for coatomer assembly. We constructed three shRNAs complementary to the three proteins, tested their knockdown efficiency, and the most effective shRNAs were selected (Figure S2A). After transfecting the shRNAs into cultured astrocytes, we measured whole-cell currents. As shown in Figure S3B–G, there was no significant change when α-COP, γ-COP, and Arf1 were independently knocked down. Thus, we conclude that the TREK1-mediated potassium current in astrocytes is only regulated by the β-COP coatomer, and not by the COP1 complex.

### 3.6. β-COP Interacts with TWIK1 in TREK1-Dependent Manner

**Figure 5:**
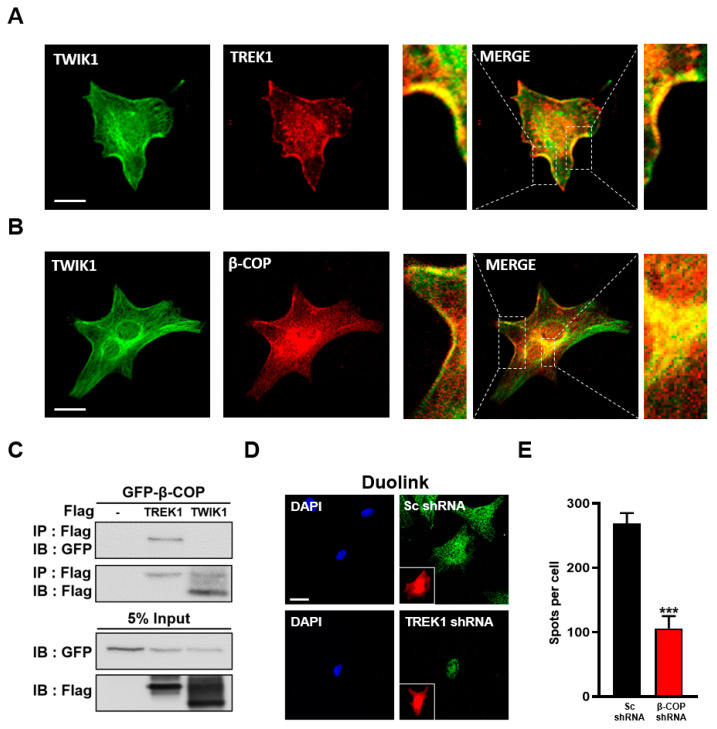
β-COP interacts with TWIK1 in a TREK1-dependent manner. (**A**) Representative immunocytochemical images of astrocytes stained with the anti-TWIK1 and anti-TREK1 antibodies. Enlargement of the merged images indicates colocalization of TWIK1 and TREK1 in the astrocyte membrane. Scale bar, 20 µm. (**B**) Representative immunocytochemical images of astrocytes stained with anti-TWIK1 and anti-β-COP antibodies. Enlargement of the merged image indicates colocalization of TWIK1 and TREK1 in the plasma membrane and inside the cell. Scale bar, 20 µm. (**C**) Co-IP in HET293T cell. After transfection of GFP-β-COP with Flag-TREK1 or Flag-TWIK1, immunoprecipitation with Flag antibody was performed and blotted with GFP antibody. (**D**) Duolink PLA assay. Intense PLA signals (green) were detected in Sc shRNA-transfected astrocytes; however, astrocytes transfected with TREK1-shRNA rarely showed PLA signals. The red fluorescent signals of mCherry protein indicated transfection of Sc shRNA or TREK1-shRNA expression vectors, which also expressed mCherry proteins as shown in Figure 4A. DAPI signals indicated nucleus of the astrocytes. Scale bar, 20 µm. (**E**) The PLA signals were counted using ImageJ software, and the average number of spots per cell is presented in the graph. The numbers on each bar indicate the n for each condition. All values are expressed as the mean ± SEM (*** *p* < 0.001). SEM, standard error of the mean. *p*-values were obtained with Student’s *t*-test.

In astrocytes, TREK1 and TWIK1 form functional heterodimeric channels in a disulfide bond-dependent manner and are predominantly localized in the cell membrane [14]. Consistently, we found that TWIK1 and TREK1 colocalized in astrocytes (Figure 5A). Since β-COP interacts with TREK1 and regulates its surface expression in astrocytes, we next tested whether β-COP also interacts with TWIK1. When anti-TWIK1 and anti-β-COP were treated used on cultured astrocytes for ICC, TWIK1 and β-COP colocalized at the astrocyte membrane (Figure 5B). To confirm that β-COP can directly bind to TWIK1, GFP-tagged β-COP (GFP-β-COP), Flag-tagged TREK1 (Flag-TREK1), or TWIK1 (Flag-TWIK1) were transfected into HEK293T cells, and a co-IP assay was performed. As a result, TWIK1 could not bind to β-COP, whereas TREK1 strongly bound to β-COP (Figure 5C). However, since TWIK1 was colocalized with β-COP in astrocytes (Figure 5B), we assumed that β-COP may be associated with TWIK1 in a TREK1 dependent manner. To this end, we evaluated the PLA signal in anti-β-COP and anti-TWIK1 treated astrocytes by performing a Duolink assay under TREK1 deficiency. In Sc shRNA-treated astrocytes, we observed strong fluorescence signals, showing an association between TWIK1 and β-COP. However, in TREK1 shRNA-treated astrocytes, weaker signals were observed (Figure 5D,E). These data suggest that TWIK1 and β-COP do not bind directly, but TWIK1 is associated with β-COP in a TREK1 dependent manner in astrocytes, in which TWIK1 and TREK1 form a heterodimer.

### 3.7. β-COP Knockdown Downregulates TWIK1 and TREK1-Mediated Currents in Astrocytes

**Figure 6:**
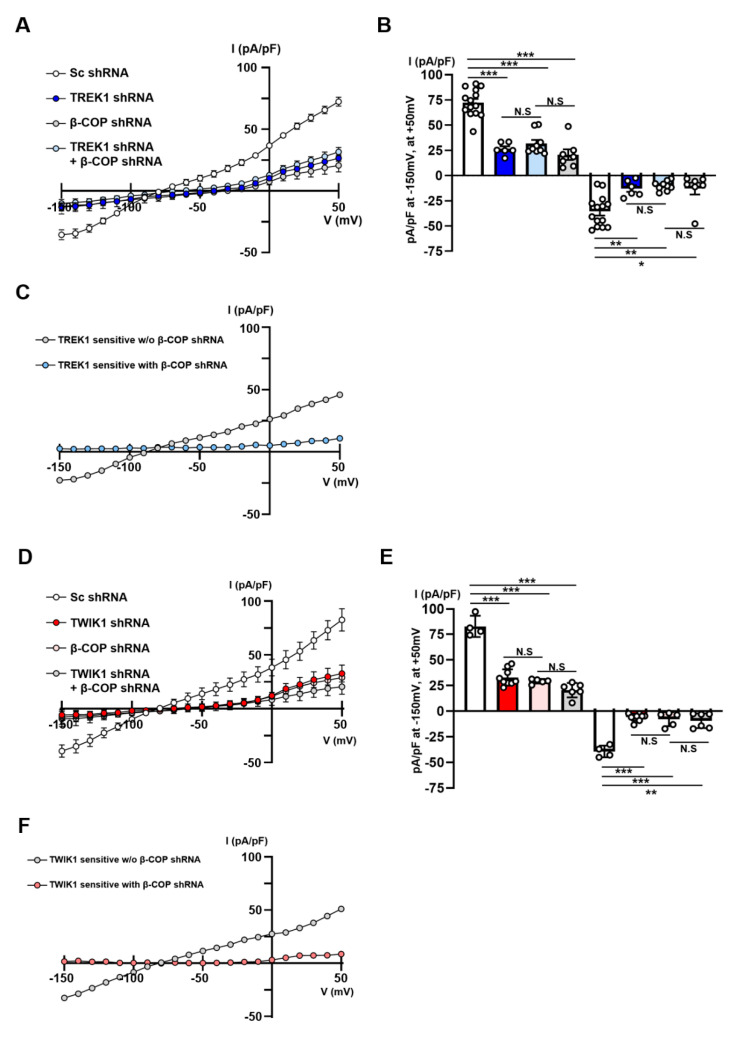
Knockdown of β-COP downregulates TWIK1/TREK1-mediated currents in astrocytes. (**A**) Average current-voltage (*I*-*V*) relationship of whole-cell K^+^ currents in cultured astrocyte transfected with Sc *shRNA*, TREK1 shRNA, β-COP shRNA, or TREK1 shRNA + β-COP shRNA. (**B**) Summary bar graph showing averaged currents at +50 mV and −150 mV in (**A**). All values are mean ± SEM. SEM, standard error of the mean. *p*-values were obtained with one-way ANOVA followed by Tukey’s post hoc test. N.S: not significant, * *p* < 0.05, ** *p* < 0.01, *** *p* < 0.001. (**C**) TREK1-sensitive currents were calculated by the difference between transfection of TREK1 shRNA without or with β-COP shRNA. (**D**) *I*-*V* relationship of whole-cell K^+^ currents in cultured astrocyte transfected with Sc *shRNA*, TWIK1 shRNA, β-COP shRNA, or TWIK1 shRNA + β-COP shRNA. (**E**). Summary bar graph showing averaged currents +50 mV and −150 mV in (**D**). All values are mean ± SEM. SEM, standard error of the mean. *p*-values were obtained with one-way ANOVA followed by Tukey’s post hoc test. N.S: not significant, ** *p* < 0.01, *** *p* < 0.001. (**F**) TWIK1-sensitive currents were calculated by the difference between transfection of TWIK1 shRNA without or with β-COP shRNA.

As TWIK1/TREK1 heterodimeric channels are essential channels responsible for linear currents in astrocytes [14], we investigated the functional association of β-COP with these channels. We utilized shRNA of TWIK1, TREK1, and β-COP to determine whether β-COP regulates currents mediated only by TREK1 or TWIK1 in astrocytes. We had previously validated TREK1 shRNA and TWIK1 shRNA [14]. To isolate only the current mediated by TREK1, we measured the current of the Sc shRNA-transfected astrocytes and then subtracted the current from TREK1 knockdown astrocytes (Figure 6A,B). Astrocytes deficient in TREK1 rarely presented a current compared to the control group. Second, to extract the current mediated by TREK1 when β-COP was knocked down, we subtracted the currents of astrocytes transfected with TREK1 shRNA and β-COP shRNA from those of astrocytes transfected with b-COP shRNA (Figure 6A,B). There was no synergistic reduction effect of the current knocked down together compared to the current knockdown of the two proteins alone. Additionally, we confirmed that the currents produced by TREK1 alone disappeared when β-COP was knocked down (Figure 6C). In summary, β-COP deficiency directly regulated TREK1-mediated currents in astrocytes. Next, to isolate only the TWIK1-mediated current regulated by β-COP in astrocytes, we conducted the test in the same way (Figure 6D,E). Consequently, as in the case of TREK1, β-COP knockdown significantly reduced TWIK1-mediated currents, and there was no synergistic effect of the TWIK1-β-COP double knockdown (Figure 6F). These results clearly suggest that β-COP is an important regulator of the TWIK1/TREK1 heterodimeric channels in astrocytes.

### 3.8. β-COP Regulates Passive Conductance in Hippocampal Astrocytes

**Figure 7:**
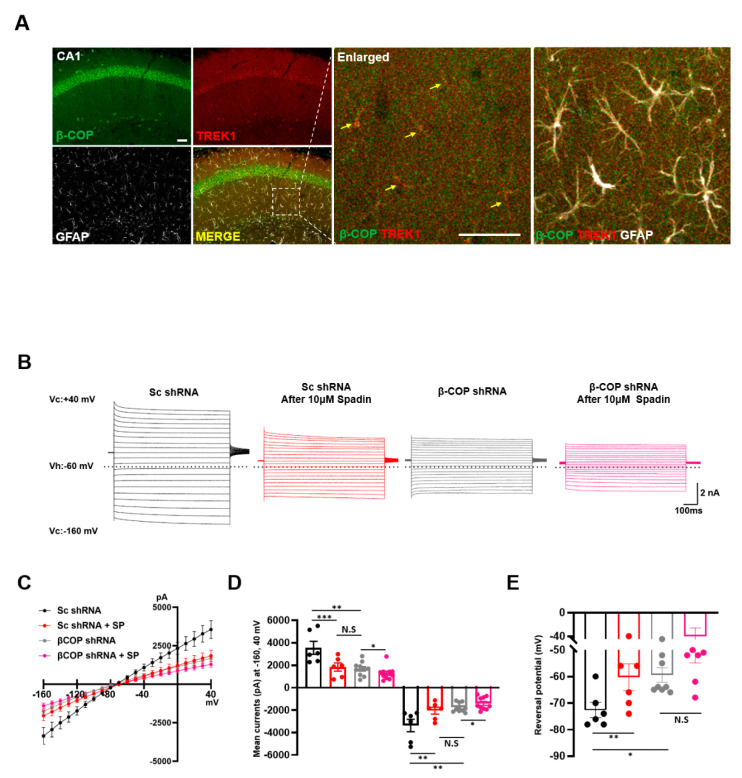
β-COP regulates passive conductance in hippocampal astrocytes. (**A**) Representative image of immunohistochemistry (IHC) in the hippocampus (CA1) and an enlarged image. β-COP (green) is expressed as abundantly as TREK1 (red) in GFAP-positive cells (white) of the hippocampus and colocalizes with TREK1 to a high degree (yellow). Scale bars, 50μm. (**B**) Representative traces of passive conductance induced by voltage stepping method in hippocampal astrocytes after injection with adeno-associated viruses expressing Sc shRNA or β-COP shRNA in the absence or presence of 10 μM Spadin. Voltages were stepped from −160 mV to +40 mV in increments of 10 mV from a holding potential of −60 mV. Dotted line indicates 0 pA. (**C**) Average current-voltage (*I-V*) relationship curves obtained from hippocampal astrocytic currents presented in (**B**). (**D**) The bar graph shows averaged current at +40 mV and −160 mV. All values are mean ± SEM. SEM, standard error of the mean. *p*-values were obtained with one-way ANOVA followed by Tukey’s post hoc test. N.S: not significant, ** *p* < 0.01, *** *p* < 0.001. (**E**) Reversal potentials also obtained from average *I-V* curves in (**C**). All values are mean ± SEM. SEM, standard error of the mean. *p*-values were obtained with one-way ANOVA followed by Tukey’s post hoc test. N.S: not significant, * *p* < 0.05, ** *p* < 0.01.

The TWIK1/TREK1 heterodimeric channel is a major molecular identity of astrocytic passive conductance in the hippocampus of adult mice [14]. To test whether β-COP merges with TREK1 in adult hippocampal astrocytes, brain slices were prepared from adult mice. IHC showed that β-COP was expressed in hippocampal (CA1) astrocytes of adult mice and was colocalized with TREK1 at high levels (Figure 7A). Next, we investigated whether β-COP regulated passive conductance in hippocampal astrocytes. To reduce the expression level of β-COP in the mouse hippocampus, an adeno-associated virus (AAV) containing a β-COP shRNA was constructed. When the AAV containing control Sc shRNA was infected, the electrophysiological recording exhibited a large passive conductance, but when β-COP was silenced by β-COP shRNA, passive conductance was dramatically reduced (Figure 7B–D). The reversal membrane potential (RMP) was depolarized from −72.7 ± 2.8 mV (Sc shRNA-infected astrocytes) to −62.5 ± 3.1 mV (β-COP shRNA-infected astrocytes) (Figure 7E). The reduction of astrocytic passive conductance by β-COP deficiency is similar to that produced by treatment with Spadin, a peptide inhibitor of TWIK1/TREK1 heterodimeric channel-mediated astrocytic passive conductance [33] (Figure 7B–D). In addition, treatment with Spadin on β-COP knockdown astrocytes reduced passive conductance (Figure 7B–D). The RMP was shifted from −72.7 ± 2.8 mV (Sc shRNA- infected astrocytes) to −60.3 ± 5.0 mV (Sc shRNA-infected astrocytes with Spadin), or −58.6 ± 3.4 mV (β-COP shRNA-infected astrocytes with Spardin) (Figure 7E). These data suggest that TWIK1/TREK1 heterodimeric channels contribute to passive conductance under β-COP deficiency. Therefore, β-COP modulates passive conductance by controlling the trafficking of TWIK1/TREK1 heterodimeric channels through protein–protein interactions with β-COP.

## 4. Discussion

This study was conducted based on previously reported experimental results that β-COP directly binds to the N-terminus of TREK1 [24]. β-COP is one of the seven subunits (α, β’, β, ε, γ, δ, ζ) of the COP1 complex that forms transport vesicles [23]. In the presence of Arf1, a small GTPase, the COP1 complex recognizes a specific sequence of target proteins, forms a complex with coatomers, and is involved in material transport [30,32]. However, our current results show that β-COP binds to TREK1 and enhances the surface expression and channel activity of TREK1 in astrocytes without the involvement of other coatomers of the COP1 complex to control surface expression of the channel (Figure 1, Figure 4 and Figure S2). These data strongly suggest that β-COP acts as a monomer to regulate TREK1 function in astrocytes. β-COP is involved in retrograde transport or anterograde transport of various channel proteins [24,34,35,36,37,38]. Therefore, β-COP-mediated channel transport may also be regulated by β-COP monomers, but not the COP1 complex. Additionally, other members of the coatomer may be able to perform transport substances as monomers similar toβ-COP. These hypotheses should be tested in future studies.

Although β-COP is involved in retrograde or anterograde transport of various channels, it remains unclear whether the amino acid sequence of target proteins causes the difference in transport direction by β-COP. In this study, we attempted to determine the exact binding site of β-COP within TREK1 to address this question. First, TREK1 in mice has two isomers with different N-terminal amino acid sequences. Therefore, we compared the interactions between the two TREK1 isoforms and β-COP, although no difference was detected (Figure 2). However, based on the qRT-PCR results, we found that the expression of TREK1 isotype 2 in astrocytes was higher than that of TREK1 isotype 1 (Figure S2) and confirmed the binding site of β-COP within the N-terminal region of TREK1 (Figure 3). The COP1 complex or β-COP is known to recognize and interact with the target protein through the following sites: 1) di-basic motif [39], 2) K(X)KXX motif [27,28] (where X represents any amino acid), 3) RXR motif [36,40], and 4) KDEL motif [41]. Using the deleted TREK1 mutants, we showed that β-COP binds to the di-lysine (KXKXX) motif at the end of the TREK1 N-terminus. Therefore, we could not find any differences in the binding motif of βCOP showing forward transport of TREK1 compared that showing retrograde transport of other channels. The relationship between the transport direction and the motif recognized by β-COP complex must be examined, comparing exact binding sites of other channels controlled by β-COP.

TWIK1 functions as a heterodimer through TREK1 and disulfide bonds in astrocytes [14]. In HEK293T cells, co-IP was performed to determine whether there was an interaction between the heterodimer members, TWIK1, TREK1, and β-COP. A strong signal was observed for the binding of TREK1 to β-COP, as in a previous study [24], but no signal was observed for TWIK1(Figure S1B). However, based on the Duolink results in astrocytes, unlike HEK293T cells, strong PLA signals were found with anti-TWIK1 and anti-β-COP, which were abolished when treated with TREK1 shRNA (Figure S1A). Thus, we revealed that β-COP does not interact with a single TWIK1. In astrocytes, where TWIK1 forms a heterodimer with TREK1, it interacts with β-COP in a TREK1 dependent manner and consequently interacts with the TWIK1/TREK1 heterodimeric channel (Figure 4). Since K2P channels mainly function as dimers [6,12], further studies are required to determine whether other single-channel binding partners interact with heterodimers containing these channels. Investigating the interaction of single-channel binding partners with multichannel complexes is essential to reveal the underlying mechanisms of protein transport. Additionally, this interaction suggests a more diverse pathway in the regulation of surface expression of membrane proteins. It also becomes an important mediator in regulating the pathological conditions associated with that channel protein without changing the number of channel proteins.

Here, we suggest that the passive conductance of astrocytes, which is mediated by a TWIK1/TREK1 heterodimeric channel, is consequently regulated by β-COP. When excess K^+^ accumulates extracellularly, the resting membrane potential changes, which greatly affects the synaptic transmission and activation of neurons [4,8,9]. Astrocytes maintain brain homeostasis by removing the excess extracellular K^+^ through K clearance, a process manifested by a linear current–voltage (*I*–*V*) relationship, that is, passive conductance [4,10]. Understanding the exact mechanism underlying this process will enable research on the various functions of astrocytes in the context of disease. β-COP regulates passive conductance through protein–protein interactions, and when β-COP is silenced in astrocytes, the membrane expression of TREK1 channels decreases (Figure 4), currents mediated by TREK1 or TWIK1 are significantly reduced, and astrocytic passive conductance is significantly reduced. Since TWIK1 mRNA expression is upregulated in the hippocampal neurons and astrocytes in the kainic acid-induced seizure mouse model [42], it seems that the increased TWIK1 expression might be a consequence of the defense mechanism against kainic acid-induced epileptic seizures. Therefore, our current study showing the β-COP-mediated increment of the TWIK1/TREK1 surface expression could help to develop therapeutic approaches for epileptic seizures.

We previously reported that TWIK1 and TREK1 among K2P channels are dominantly expressed in hippocampal astrocytes and the hippocampal astrocytic passive conductance is reduced by the deficiency of TWIK1 or TREK1 but not by the deficiency of TREK2 [14]. However, it has been reported that the expression patterns of K2P channel are regionally dependent in the brain and other K2P members such as TREK2 or TASK1 are also expressed in astrocytes [6]. In addition, TWIK1 can form heterodimeric channels with TREK2 or TASK1 in a heterologous expression system [14]. Therefore, it is also plausible that astrocytic K^+^ conductance in diverse regions of the brain might be controlled by different K2P channels. In addition to K2P channels, inwardly rectifying Kir4.1 channels in astrocytes also can be involved in passive conductance of hippocampal astrocytes. Since we previously found that deficiency of Kir4.1 also slightly reduced passive conductance of hippocampal astrocytes and significantly depolarized RMP of astrocytes [14], it is also possible that β-COP also regulates the surface expression of Kir4.1 channels in hippocampal astrocytes. In addition, it is also worthwhile to examine the specific roles of K2P channels and Kir4.1 channels in various region-specific astrocytes.

In conclusion, we reveal for the first time that an important characteristic of astrocytes, passive conductance, is regulated through the protein–protein interactions with β-COP. β-COP is an important binding protein capable of modulating passive conductance mediated by the TWIK1/TREK1 heterodimeric channel. Therefore, these data suggest a new molecular structural mechanism for passive conductance. This research advanced our understanding of the mechanisms that regulate K^+^ clearance in astrocytes and the various regulatory pathways of TWIK1/TREK1 heterodimeric channels promoted by their binding proteins.

## Data Availability

All data will be available upon reasonable request by emailing the corresponding author.

## Supplementary Materials

The following supporting information can be downloaded at: https://www.mdpi.com/article/10.3390/cells11203322/s1, Figure S1: Relative expression of TREK1 isoforms in Astrocytes; Figure S2: Other COP1 subunits do not affect the K^+^ current in astrocytes.
