# Supplementary material for: β-COP Regulates TWIK1/TREK1 Heterodimeric Channel-Mediated Passive Conductance in Astrocytes
Source: Cells. 2022 Oct 21;11(20):3322. doi: 10.3390/cells11203322 (PMC9600989; doi:10.3390/cells11203322)
Supplement: Supplementary file 1 [file cells-11-03322-s001.zip › cells-1959073-SM.pdf]

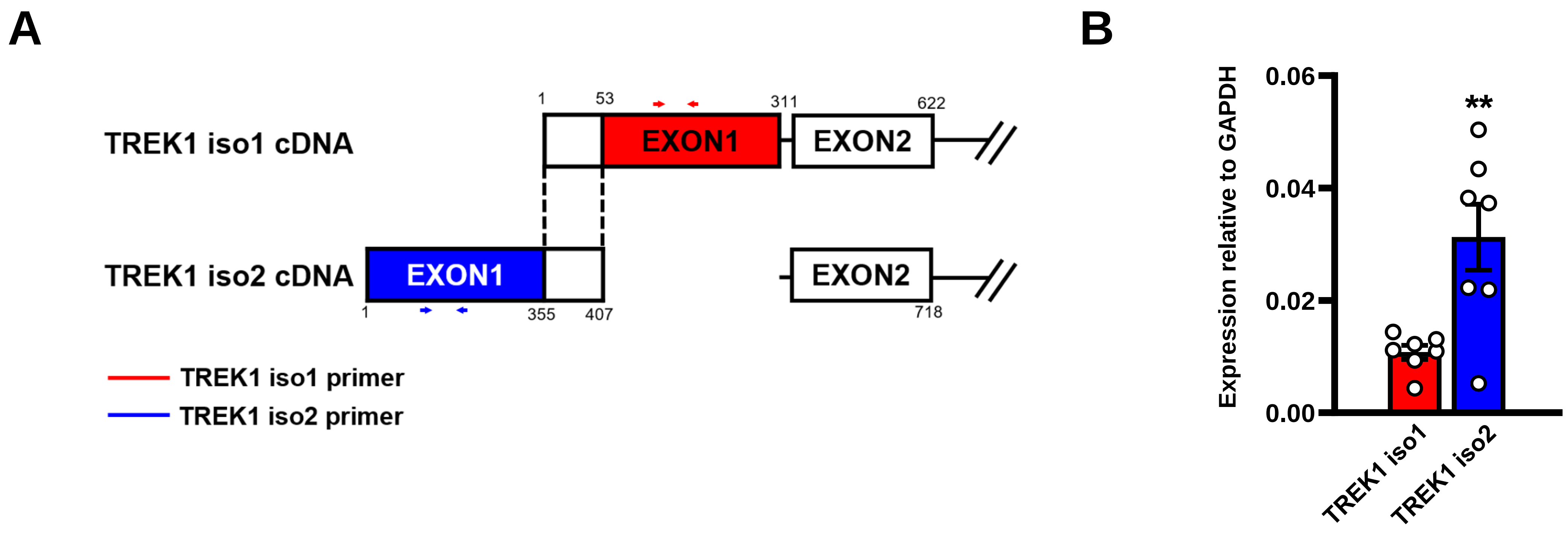

Figure S1. Relative expression of TREK1 isoforms in Astrocytes. (A) Schematic diagram of the cDNA for each TREK1 isoform. Primers for qRT-PCR, indicated by small arrows, were designed in exon1 of each isoform. (B) Bar graphs are qRT-PCR results using each primer. Normalized with GAPDH(n=7). All values are mean ± SEM (**$p < 0.01$). qRT-PCR, quantitative reverse transcription polymerase chain reaction; SEM, standard error of the mean; TWIK1, tandem of pore domains in a weak inward rectifying K^+ channel; TREK1, TWIK-related K^+ channel 1; GAPDH, glyceraldehyde 3-phosphate dehydrogenase.

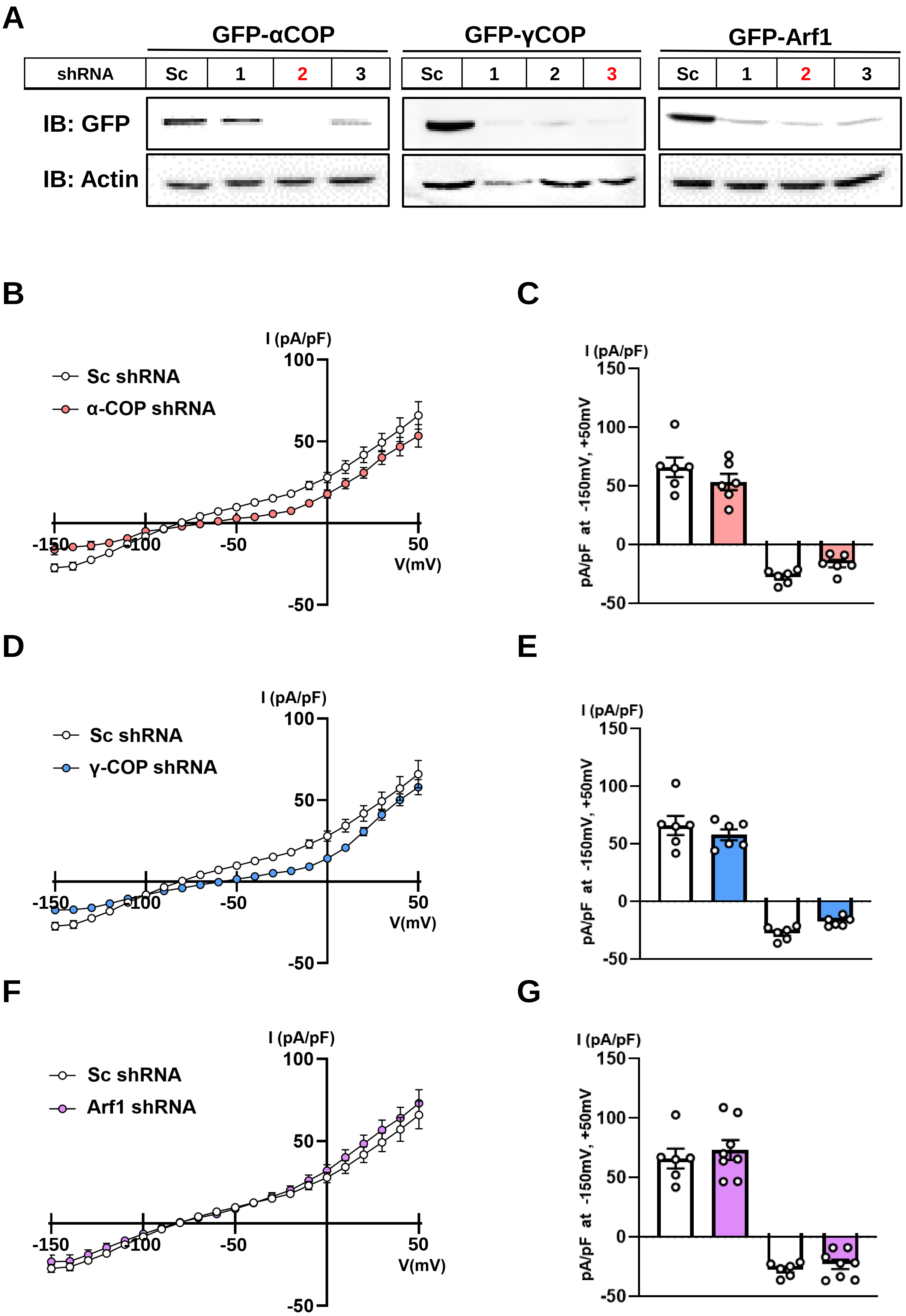
A
GFP-αCOP
GFP-γCOP
GFP-Arf1
shRNA
Sc
1
2
3
IB: GFP
IB: Actin
B
I (pA/pF)
Sc shRNA
α-COP shRNA
V(mV)
C
I (pA/pF)
pA/pF at -150mV, +50mV
D
I (pA/pF)
Sc shRNA
γ-COP shRNA
V(mV)
E
I (pA/pF)
pA/pF at -150mV, +50mV
F
I (pA/pF)
Sc shRNA
Arf1 shRNA
V(mV)
G
I (pA/pF)
pA/pF at -150mV, +50mV

Figure S2. Other COP1 subunits do not affect the K^+ current in astrocytes. (A) α-COP, γ-COP, Arf1 shRNA validation test in HEK293T cells. GFP-tagged α-COP, γ-COP, and Arf1 were co-transfected with each of three specific shRNAs. The most efficient shRNA (red) was used in the experiment. (B), (D), (F) Average current-voltage (I-V) relationship of Sc shRNA (white), α-COP shRNA (light orange), γ-COP shRNA (light sky), and Arf1 shRNA (light violet) transfected astrocytes. The voltage ramp-induced whole-cell current traces were recorded from each cell. (C), (E), (G) Bar graph at -150 mV and +50 mV in (B), (D), (F). All values are mean $\pm$ SEM (***$p < 0.001$). SEM, standard error of the mean; COP, coat protein; COP1, coat protein complex 1; Arf1, ADP-ribosylation factor 1; TWIK1, tandem of pore domains in a weak inward rectifying K^+ channel; TREK1, TWIK-related K^+ channel 1; GFP, green fluorescent protein; Co-IP, co-immunoprecipitation; Sc, scrambled; HEK, human embryonic kidney.
